# Opioid-Free Anaesthesia Reduces Complications in Head and Neck Microvascular Free-Flap Reconstruction

**DOI:** 10.3390/jcm12206445

**Published:** 2023-10-10

**Authors:** Paulo-Roberto Cardoso Ferreira, Rita Isabel Pinheiro De Oliveira, Marta Dias Vaz, Carla Bentes, Horácio Costa

**Affiliations:** 1 Centro Hospitalar de Vila Nova de Gaia, 4434-502 Vila Nova de Gaia, Portugal; 2 Medical Sciences Department, University of Aveiro, 3810-193 Aveiro, Portugal

**Keywords:** Opioid-Free Anaesthesia, multimodal anaesthesia, fluid management, free flap, reconstructive surgery, head and neck reconstruction, postoperative complications

## Abstract

Head and neck free-flap microvascular surgeries are complex and resource-intensive procedures where proper conduct of anaesthesia plays a crucial role in the outcome. Flap failure and postoperative complications can be attributed to multiple factors, whether surgical- or anaesthesia-related. The anesthesiologist should ensure optimised physiological conditions to guarantee the survival of the flap and simultaneously decrease perioperative morbidity. Institutions employ different anaesthetic techniques and results vary across centres. In our institution, two different total intravenous approaches have been in use: a remifentanil-based approach and a multimodal opioid-sparing approach, which is further divided into an opioid-free anaesthesia (OFA) subgroup. We studied every consecutive case performed between 2015 and 2022, including 107 patients. Our results show a significant reduction in overall complications (53.3 vs. 78.9%, *p* = 0.012), length of stay in the intensive care unit (3.43 ± 5.51 vs. 5.16 ± 4.23 days, *p* = 0.046), duration of postoperative mechanical ventilation (67 ± 107 vs. 9 ± 38 h, *p* = 0.029), and the need for postoperative vasopressors (10% vs. 46.6%, *p* = 0.001) in the OFA group (vs. all other patients). The multimodal and OFA strategies have multiple differences regarding the fluid therapy, intraoperative type of vasopressor used, perioperative pathways, and various drug choices compared to the opioid-based technique. Due to the small number of cases in our study, we could not isolate any attitude, as an independent factor, from the success of the OFA strategy as a whole. Large randomised controlled trials are needed to improve knowledge and help define the ideal anaesthetic management of these patients.

## 1. Introduction

Microvascular free tissue reconstruction (FTR) is considered the best option for reconstructing large facial defects. Free-flap surgeries for head and neck reconstruction are complex and resource-intensive procedures, requiring coordinated multidisciplinary teams and being one of the most expensive solid malignancies to treat [1,2]. The likelihood of postoperative complications and a greater length of stay (LOS) in hospital is higher than it is with most surgical procedures [3,4]. In recent years, research regarding intraoperative options and care pathways has increased in an attempt to improve clinical and surgical results as well as effectively managing resources in order to reduce their burden on health systems [5,6,7,8]. At our institution, cancer extirpation and neck dissection are followed by immediate reconstruction in the same operating time. The flap is harvested simultaneously by part of the surgical team.

The conduct of anaesthesia itself is challenging due to the difficult airway, long duration of surgery, and massive surgical resection with immense haemorrhage and fluid shifts [5,9,10]. Balanced general anaesthesia (BGA) is the most common technique used in anaesthesia care. There is overwhelming evidence that this approach uses less of each drug than if the drugs were administered alone [11,12,13]. The conduct of anaesthesia itself is challenging due to the difficult airway, long duration of surgery, and massive surgical resection with immense haemorrhage and fluid shifts. To date, BGA has relied almost exclusively on opioids to manage intraoperative nociception and postoperative pain. Nociception induced by surgery is the primary reason for placing a patient under anaesthesia. If uncontrolled, nociception is the main source of intraoperative haemodynamic and stress responses and postoperative chronic pain syndromes. Opioids are the most effective antinociceptive agents; however, overreliance on them exposes their adverse effects, including nausea, vomiting, constipation, ileus, pruritus, and respiratory depression [12]. Opioid-sparing strategies improve outcomes in various dimensions of intraoperative and postoperative care. In the last decade, opioid-free anaesthesia (OFA) research has gained traction but there is still a great debate over its feasibility, safety, and efficiency [11,12,14]. More research is needed to identify the rationale for the ideal combination of drugs and if opioid use should only be reduced or completely avoided.

There is limited published evidence, namely, large series or randomised controlled trials, for the ideal anaesthetic management in FTR procedures because of their relative rarity in comparison to other types of surgery [2,5,15]. As of 2015, our institution’s Maxillofacial and Reconstructive Surgery Anaesthesia team implemented an updated multimodal anaesthetic (MMOD) protocol for free-flap head and neck reconstruction surgery. This protocol was developed while addressing some challenges of the standard remifentanil-based (REMI) intraoperative analgesia and restrictive fluid approach, changing it to a multimodal opioid-sparing analgesia strategy with goal-directed fluid therapy and early weaning of mechanical ventilation [1,4,7,16]. MMOD protocol considered the option of excluding remifentanil and using vasodilators to achieve controlled hypotension, resulting in the OFA subgroup. Due to the paucity of quality data in this field, care differs between institutions and even practitioners. Some aspects of the protocol were founded on extrapolated evidence from other major surgical procedures and focused on Enhanced Recovery After Surgery principles [2,4].

Both the classic approach (REMI) and the new multimodal protocol (MMOD) could be chosen depending on the anaesthetist’s preference and experience. We have collected data from every patient submitted to major head and neck reconstruction surgery performed at our institution between 2015 and 2022 and a 1-year follow-up and compared relevant outcomes.

## 2. Material and Methods

This study is a single-centre cohort and was conducted at Centro Hospitalar de Vila Nova de Gaia/Espinho (CHVNG/E), Portugal and approved by the institutional ethics committee. This manuscript adheres to the Strengthening the Reporting of Observational Studies in Epidemiology (STROBE) statement [17]. After obtaining signed consent, patients scheduled for major free-flap head and neck reconstructive surgery were enrolled. Exclusion criteria included patients submitted to reconstructive surgery other than head and neck and surgeries that did not include a free flap with microvascular anastomosis reconstruction.

A total of 107 patients were included in this study, with surgical interventions occurring between July 2015 and July 2022 and follow-up data being gathered in August 2023. Although not randomly assigned ab initio, patient distribution occurred in a quasi-random manner since it depended solely on the schedule of the Anaesthesia Department without prior knowledge of the daily operating plan. The criteria of inclusion in this cohort were: patients being submitted to (a) major maxillofacial procedures for head and neck cancer, radiotherapy side-effects, or trauma and those involving (b) immediate reconstruction using a free flap with arterial and venous cervicofacial anastomosis in the same operative time.

All data were collected from sources including the electronic anaesthetic and surgical reports from the operating room (OR), Intensive Care Unit (ICU) and Plastic Surgery Ward (PSW) clinical records and 1-year follow-up consultations. Demographics and preoperative clinically relevant data were gathered from pre-anaesthesia evaluation records and perioperative analytic results extracted directly from lab data.

Reported blood loss was recovered from anaesthesia records. This value resulted from an estimation performed at the end of the procedure by the surgical and anaesthetic teams, relying on the contents of the suction system, gauze’s and sponges’ subjective evaluation, and other visible blood losses. Due to logistic limitations, surgical gauzes and covering tissues are not consistently weighted. As described in the literature, this method is not reliable and frequently underestimates effective blood loss [18].

Correct blood and fluid management should take into account adequate estimated “visible” blood losses but also “hidden” blood losses. Since the first might be compromised and the second is, per definition, unmeasurable, we used the hemogram results from blood collected 12 h after ICU admission, as a surrogate for Hb_Nadir_, to estimate the effective haemoglobin mass deficit [18]. The volaemia calculation was performed using Nadler’s method and an estimation of effective blood loss was conducted using the Haemoglobin Mass Loss method described by Jaramillo et al. [18]. Both reported and estimated haemorrhages were used for comparison.

### 2.1. Anaesthesia Management Strategy

The anaesthesia technique was assigned to remifentanil-based (REMI) analgesia or multimodal (MMOD) analgesia, depending on the intraoperative usage of two or more drugs with different mechanisms to achieve analgesia and sparing opioid administration. Contained within the multimodal group, an opioid-free (OFA) subgroup—including only patients who did not receive any opioids during surgery—was also independently analysed. The total intravenous anaesthesia technique with propofol was performed in all cases. Target-controlled-infusion (TCI) algorithms or defined-rate syringe pumps were used and parameters were titrated to obtain an adequate depth of anaesthesia, using a bispectral index (BIS) between 40 and 60.

Data regarding fluid therapy (type of solution and quantity, including blood products) were obtained from the electronic anaesthesia record software (B-Anesthesic and PatientCare, B-Simple, Porto, Portugal). Fluid losses, such as blood loss, urine output, and “insensitive” loss (including evaporation, transudation, and basal metabolic requirements), were recorded. Rates were subsequently calculated in mL/kg and mL/kg/h using the recorded surgical duration. Net fluid balance (NFB) was the result recorded; it was automatically calculated by the electronic record software. Information on the use of sympathomimetics in the intraoperative period and ICU was extracted from medical records. Due to missing data (rate changes, total amount, and number of boluses) in the electronic records, these drugs were grouped as vasopressors (phenylephrine, ephedrine, epinephrine) or cardiotonic (dopamine and dobutamine) and were transformed into a dichotomous variable.

Postoperative analgesia administered in the ICU, not at the discretion of the anaesthetist but influenced by the intensive care team, was independently allocated and compared. Postoperative uncontrolled pain was defined as more than one reference to moderate or severe pain (>3 in the numeric pain scale) that did not subside with the prescribed analgesia.

### 2.2. Surgical Data and Outcomes

Surgeries were grouped by the reason that motivated the procedure (cancer, radionecrosis, or trauma), type of surgery (tumour removal, neck dissection, protective tracheostomy), and free-flap donor site [Antero-Lateral Tigh (ALT), Radial and Ulnar Forearm (FRA), Iliac Crest (ICR), Fibular (FIB), Other (OTH)]. The surgical site, history of surgery, current tumour recurrence, and prior radiation exposure were independently considered.

Outcomes included early surgical complications (cervical haematoma, active identifiable blood leak from vessels, and mechanical failure of the anastomosis), late receptor site complications (surgical site infections, flap ischaemia after >48 h documented viability), and medical complications (pneumonia, other non-surgical infections, uncontrolled pain, analgesia-attributable adverse effects). Overall complications were classified according to the Clavien-Dindo classification, graded 1 to 5 [19].

Reconstruction success was defined in five levels: 0 = integrated flap, 1 = minor defects that subsided with a conservative approach, 2 = small defects that needed minor surgical intervention (re-suture, small partial-thickness skin grafts), 3 = partial necrosis of the flap requiring debriding and reconstructive surgery, and 4 = flap failure and removal due to extensive necrosis within 30 days (Table 1).

The surgery duration, LOS in the ICU and Intermediate Care Unit, and duration of hospitalisation (DoH) were obtained from official administrative records. The duration of invasive mechanical ventilation (IMV), time to enteral nutrition tolerance, start of tracheal cannula clamp training, and cannula removal were obtained from clinical records when applicable.

### 2.3. Statistical Methods

Associations among the occurrence of perioperative complications with the variables in the data set assumed binomial distribution. Chi-square and Fisher’s exact tests were used to examine associations between the outcome and all dichotomous variables. Univariate models for failure were considered. Continuous variables were checked for normality using the Shapiro–Wilk test and the Student’s *t*-test was applied to investigate the association between the outcome and normally distributed variables. Two pairs of groups were created for comparison. All *p*-values shown under MMOD result from comparing MMOD and REMI; the *p*-values under OFA compare OFA and all others.

Multivariable adjustment was performed, using forward stepwise binary logistic regression for overall complications and flap loss. The variables considered were predetermined and included anaesthetic strategy (OFA vs. all others), demographics, ASA status, FTP donor site, reported blood loss, fluid therapy, and the comorbidities, as listed in Table 2.

Due to our limited sample and non-random comparisons, we abstained from extensive statistical testing and focused on the detailed description of our treatment group’s characteristics and prognosis.

## 3. Results

Opioid-based anaesthesia management, using REMI, was applied to 54.2% of patients (*n* = 58). The MMOD protocol was employed in 45.8% of patients (*n* = 49). Thirty patients (28.0%) underwent opioid-free anaesthesia (OFA), which formed almost 2/3 of the MMOD group.

### 3.1. Type of Surgery

Among the 107 patients, the most common indication for reconstructive surgery was cancer *n* = 82 (76.6%), followed by radiation necrosis *n* = 21 (19.6%), and trauma *n* = 4 (3.8%). In 28 patients (26.2%), the relapse of a previously treated cancer was the reason for surgery. Almost half of the patients (*n* = 49, 45.8%) had a history of head and neck surgical procedures and 32 patients (29.9%) underwent prior radiation therapy. Surgical failure was associated with radionecrosis as the surgical indication (*p* < 0.05) (Table 1).

FTR donor-site choice depended on the characteristics of the defect and the patients. Four main types of donor sites were identified: 39 fibular osteocutaneous (36.5%), 26 radial and ulnar forearm (14.1%), 17 Iliac crest (15.9%), and 15 anterior lateral thigh (14.0%). Uncommon donor sites (e.g., temporoparietal, rectus abdominis) were verified for FTR and were used in 10 cases (9.3%). Uncommon flap donor sites had higher flap-loss rates (20%), followed by fibular osteocutaneous (12.8%); however, none were independently associated with the outcomes (Table 3).

The mean surgical time was 601 min in the flap failure group (*n* = 10, 9.3%) versus 563 min in the non-flap failure group; however, this was not statistically significant.

### 3.2. Patient Demographics

All patients were caucasian, 64.5% were male (*n* = 69) and 35.5% female (*n* = 38). Males were significantly more prone to develop pneumonia (*p* = 0.002) and overall complications (*p* = 0.026). The mean age at the time of surgery was 60 years. Regarding the American Society of Anesthesiology (ASA) classification, 5.6% were classified as ASA class 1 (*n* = 6), 51.4% as ASA class 2 (*n* = 55), and the remaining 43% as ASA class 3 (*n* = 46). Demographics and comorbidities are reported in Table 4.

The risk factors associated with flap loss were the BMI < 18 class (*p* = 0.04), ASA > 2 (*p* = 0.044), diabetes (*p* = 0.001), COPD (*p* = 0.001), lower preoperative platelet count (*p* = 0.000), and preoperative antiplatelet medication (*p* = 0.019). Pneumonia was associated with the male sex (*p* = 0.002), absence of hyperlipidaemia (*p* = 0.025), and lower preoperative weight (*p* = 0.041)—but not with BMI < 18 class. Only the male sex (*p* = 0.026) and high preoperative INR value (*p* = 0.045) were associated with overall complications (Table 4).

### 3.3. Postoperative Complications

Complications were identified in 71.4% (*n* = 76) of the procedures, encompassing surgical and non-surgical ones. Early surgical complications—defined as those arising in the first 48 postoperative hours—usually related to inadequate flap perfusion, active haemorrhage, or significant haematoma, were identified in 21.4% of the procedures (*n* = 23). Thirteen (56.5%) of those underwent reoperation within the first 48 h. The remainder were approached conservatively.

Delayed surgical complications, defined as those arising after 48 h, such as infection, dehiscences, or flap failure, were identified in 29.9% (*n* = 32) of procedures. Partial necrosis was reported in 11.2% of procedures (*n* = 12). Delayed complications resulted in re-interventions within 30 days of the primary surgery for 78.5% of patients (*n* = 25).

The most common non-surgical complications were analgesia-related (obstipation, prolonged digestive stasis, and delirium), occurring in 43.0% (*n* = 46) of all patients. Pneumonia was identified in 21.5% (*n* = 23) of patients and other types of infections were found in 14.0% (*n* = 15).

The presence of an abundance of tracheal secretions, without respiratory distress nor radiological evidence of pneumonia, but implicating cough assist and/or regular suction, was identified in 11.2% (*n* = 12) of patients. It was considered a ventilation-related complication. Most of the patients identified as having any complication had more than one system affected.

Survival after 1 year was 86.9% (*n* = 93). Four patients (3.8%) died during hospitalization, averaging 42.0 ± 17.1 days after surgery. Three had complications not directly related to the surgery and one developed a surgical-site infection complicated with bacteriemia and sepsis (Table 5).

### 3.4. Blood Loss

The intraoperative reported blood loss averaged 757 mL, with no statistically significant differences between REMI, MMOD, or OFA. The reduction in blood haemoglobin concentration 12 h after the end of surgery (Hb_preop_ − Hb_nadir_) averaged 4.39 ± 1.95 g/dL; the OFA strategy resulted in a significantly lower Hb drop (3.61 ± 2.18 g/dL, *p* = 0.001).

The haemoglobin mass loss (HbML, in grams) during the intraoperative period was defined as volaemia × [Hb_preop_ − Hb_nadir_] [18]. Each unit of packed red blood cells (RBCs) transfused in the OR added 43 g to HbML [18]. HbML in the OR averaged 197.5 g, which resulted, by unit conversion to volume, in a haemoglobin-related calculated blood loss (HbCalc) averaging 1664 mL. The HbCalc is almost double the operating team’s estimative value for blood loss. The REMI group had lower estimates of blood loss (649 ± 368 mL) but greater underestimation compared to HbCalc (1642 ± 653 mL). In the MMOD protocol, the estimation of blood loss was 886 ± 619 mL, which is closer to the HbCalc (1669 ± 749 mL, *p* = 0.011) (Table 6).

The flap failure group had fewer blood losses, both estimated and calculated, via HbCalc (1586 mL vs. 1672 mL); none were statistically relevant to the outcome.

The transfusion of blood products in the OR occurred in 34.5% of patients (*n* = 37), averaging 3.6 units transfused per patient (2.3 units RBC, 1.1 fresh frozen plasma, and 0.2 platelet pool). In the ICU, 35.5% of patients (*n* = 38) were transfused, averaging 2.3 units RBC per patient. The REMI group preferred fewer and later transfusions (OR 0.48 uRBC, ICU 0.84 uRBC, Total 1.33 uRBC) compared to the MMOD group (OR 1.26 uRBC, ICU 0.78 uRBC, Total 2.04 uRBC) and the OFA subgroup (OR 1.80 uRBC, ICU 0.70 uRBC, Total 2.50 uRBC) (Table 6).

Blood transfusions in the OR and ICU were not significantly different between the flap-loss group (OR 0.9 ± 2.3 uRBC, ICU 1.0 ± 2.3 uRBC) and the successful surgery group (OR 0.8 ± 1.5 uRBC, ICU 0.8 ± 1.8 uRBC).

### 3.5. Fluid Management and Net Fluid Balance

Fluid administration in the OR averaged 6784 mL; however, there were significant differences (*p* < 0.01) between the REMI group (6099 mL, 8.50 mL/kg/h) and the MMOD group (7596 mL, 9.96 mL/kg/h).

Overall fluid losses (FLs) in the cohort averaged 5019 mL and NFB was positive (+1808 mL). Significant differences were found between the anaesthetic approach and the results of the FL estimation between the REMI group (FL 3907 mL, NFB +2228 mL), MMOD group (FL 6335 mL, NFB +1311 mL, *p* = 0.001), and OFA subgroup (FL 7573 mL, NFB +344 mL, *p* = 0.001) (Table 6).

The mean urine output (overall 2.56 mL/kg/h) was lower in the REMI group 2.10 mL/kg/h compared to MMOD 3.10 mL/kg/h and OFA 3.23 mL/kg/h (*p* > 0.10).

The estimation for other fluid losses, excluding blood and urine, in the cohort was FL_Other_ = 2351 mL (3.51 mL/kg/h). The REMI group consistently estimated lower in FL_Other_ 1697 mL, 2.53 mL/kg/h when compared to MMOD FL_Other_ 3125 mL, 4.66 mL/kg/h, (*p* < 0.01) and OFA FL_Other_ 4237 mL, 6.38 mL/kg/h, (*p* < 0.01). This result is largely influenced by the number of patients with zero mL considered in other losses [overall *n* = 37 (34.5%); REMI *n* = 24 (41.4%); MMOD *n* = 13 (26.5%); OFA *n* = 1 (3.3%)].

Flap-loss patients had a greater fluid administration rate (10.64 mL/kg/h vs. 9.01 mL/kg/h) but, paradoxically, a less positive net fluid balance (NFB) (+1549 mL vs. +1835 mL); none of these factors achieved statistical significance (*p* > 0.30, *p* > 0.20, respectively). When accounting for HbCalc for the NFB calculation, a lower NFB was seen in the flap failure group (+728 mL vs. +930 mL); this was still not statistically significant (Table 6).

### 3.6. Sympathomimetic Drugs

Differences in sympathomimetic drug use were also observed. The REMI group administered vasopressors (VPr) in 43% of cases (*n* = 25) and inotropes (InT) in 5% of cases (*n* = 3). The MMOD group opted for VPr in 20% of patients (*n* = 10) and InT in 30% (*n* = 15). Regarding the OFA subgroup, VPr were administered in 27% of patients (*n* = 8) and InT in 37% of cases (*n* = 11). Intraoperative vasoactive drugs were used in 40% of flap failure cases and 51% of flap survival cases were treated with sympathomimetics in the OR. Vasopressor support in the ICU was observed in 34.6% of patients (Overall *n* = 37), REMI *n* = 27 (46.6%), MMOD *n* = 10 (20.4%, *p* < 0.01), OFA *n* = 3 (10%) *p* < 0.01]. Flap necrosis was associated with ICU vasopressor use (70% vs. 31%, *p* < 0.02) but not with OR use (Table 6).

### 3.7. Postoperative Invasive Mechanical Ventilation

The increased postoperative duration of IMV_ICU_ was not associated with flap loss (70 ± 72 h vs. 48 ± 99 h, *p* = 0.097) but was an independent factor for pneumonia (87 ± 139 h vs. 41 ± 80 h, *p* = 0.043). The time to restoration of the spontaneous ventilation (SpVent) was significantly different between the three groups. In the REMI group, only 22.4% of patients (*n* = 13) were admitted into the ICU in SpVent; whereas, in the MMOD group, 69.4% (*n* = 34, *p* < 0.01) of patients were admitted in SpVent. Only one patient in the OFA subgroup was mechanically ventilated upon ICU admission, meaning SpVent occurred in 96.7% of cases (*n* = 29, *p* < 0.01).

The IMV duration in the ICU (IMV_ICU_) was 66 ± 97 h in the REMI group and this was almost halved in the MMOD group (33 ± 95 h, *p* > 0.05). OFA patients had a seven-fold reduction in IMV_ICU_ duration (9 ± 38 h), which was statistically significant (*p* < 0.03).

A significant reduction in the time required to start tracheal cannula occlusion training (*p* < 0.04), and the ability to tolerate complete occlusion (*p* < 0.02), was associated both with the MMOD and OFA strategies (Table 7).

### 3.8. Pain and Analgesia

Only 77% of all of the procedures had other minor analgesic drugs (e.g., acetaminophen, metamizole, NSAIDs) administered in the OR (REMI 64%, MMOD 92%, OFA 97%). The postoperative analgesia strategy was strongly correlated with the anaesthetic strategy group (*p* < 0.01) (Table 8).

Moderate to severe pain occurring in the ICU (P-ICU) and pain occurring in the plastic surgery ward (P-PSW) was more prevalent in the REMI group [P-ICU *n* = 9 (16%), P-PSW *n* = 9 (16%)] than in the MMOD group [(P-ICU *n* = 6 (12%), P-PSW *n* = 4 (8%)] and OFA subgroup [(P-ICU *n* = 2 (7%), P-PSW *n* = 2 (7%)]; however, this did not achieve statistical significance. Analgesia-related complications (ARC) were also more prevalent in the REMI group with 35 patients (60%) when compared to MMOD *n* = 22 (45%, *p* > 0.10). he TOFA patients had a significantly lower number of patients suffering from ARC (*n* = 7, 23%, *p* < 0.01).

Postoperative analgesia with fentanyl perfusion yielded the worst results (*n* = 34, P-ICU 25%, P-PSW 17%, ARC 88%) when compared to continuous tramadol perfusion (*n*= 34, P-ICU 9%, P-PSW 12%, ARC 53%) and ketamine-midazolam patient-controlled analgesia (PCA-KM) (N = 31, P-ICU 0%, P-PSW 3%, ARC 19%). Other strategies resulted in an intermediate outcome (N = 18, P-ICU 33%, P-PSW 22%, ARC 67%). None were independent predictors due to the small numbers of each (Table 9).

### 3.9. Length of Stay in the ICU and Duration of Hospitalization

An association between the implemented anaesthetic protocol and ICU_LOS_ and DoH in days was observed. The REMI group had a mean ICU_LOS_ of 5.31 ± 4.74 days and DoH of 27.1 ± 17.0 days; the MMOD ICU_LOS_ was 3.92 ± 3.06 days (*p* > 0.05) and the DoH was 24.0 ± 13.7 days (*p* > 0.30). OFA patients, versus all others, had a statistically significant reduction of almost 2 days, averaging an ICU_LOS_ of 3.43 ± 3.51 days (*p* < 0.05), and was short of reaching significance for a 3-day reduction regarding the DoH 24.1 ± 13.1 days (*p* > 0.50) (Table 10).

### 3.10. Other Factors

Measurable differences in outcomes were associated with the anaesthetist involved; however, when including the impact of the chosen technique in the outcome, those differences were mitigated.

Rocuronium administration was significantly higher in the REMI (0.373 ± 0.170 mg/kg/h) and the MMOD groups (0.379 ± 0.206 mg/kg/h) compared to the OFA subgroup (0.295 ± 0.123 mg/kg/h, *p* < 0.01). Rocuronium administration was not independently associated with measured outcomes.

The time to the re-establishment of gastrointestinal function after ICU admission was decreased in the MMOD (46 ± 55 h, *p* > 0.10) and OFA groups (41 ± 56 h, *p* = 0.06) compared to REMI (60 ± 35 h) (Table 9).

The intraoperative uses of antifibrinolytic agents, antiplatelet drugs (acetylsalicylate), hydroxyethyl starch, gelatin, albumin, or preemptive analgesia were significantly different between the REMI and MMOD approaches but did not achieve significance in any of the measured outcomes. The use of heparin was associated with increased flap failure (36.4%) compared to non-administration (6.3%, *p* < 0.02). It also increased overall complications, identified in 81.8% of patients treated with heparin versus an incidence of 69.8% in the others (*p* < 0.04) (Table 11).

### 3.11. Multivariate Analysis

The binary logistic regression model for overall complications excluded all variables, except OFA (OR 0.30, CI 95% 0.04–0.89, *p* = 0.011) and radionecrosis (OR 5.3, CI 95% 1.12–25.41, *p* = 0.035)., as reason for surgery The regression model for flap necrosis only included BMI (OR 0.76, CI 95%, 0.582–0.995, higher BMI protects), radionecrosis (OR 4.63, CI 95% 0.97–21.95), and ASA status ≥ 3 (OR 11.77, CI 95% 1.76–78.82) (Table 2).

## 4. Discussion

Complications requiring surgery (31.8%) and failure rates (9.3%) in our cohort were consistent with previously reported rates [6,20,21]. Surgical failure was associated with radionecrosis as the surgical indication. Uncommon flap donor sites performed the worst (nasolabial flap, and gracilis, both lost), followed by FIB, as reported by other authors [8,10,22]. Surgical site conditions, such as a history of radiation exposure and prior cervical surgery, negatively affected the outcome, which is consistent with published evidence. Mean surgical time (567 min) in our cohort is higher than in other series [9,23,24,25], possibly due to a cancer extirpation and neck dissection being performed in the same surgical time as a reconstruction, as opposed to deferred reconstruction observed in other series. This has predictable implications on other variables, such as blood loss, complication rates, and LOS in the ICU and total DoH.

Our study found that a multimodal anaesthetic strategy is associated with improved outcomes, including the overall surgical result, incidence of surgical and medical complications, and reduced adverse effects attributable to analgesic drugs, consistent with other authors [12]. Both MMOD and OFA, but especially OFA, significantly reduced the IMV_ICU_ duration, pneumonia incidence, ICU_LOS_, time to the reestablishment of gastrointestinal function, time to tracheal decannulation, and DoH. The reduction in the ICU_LOS_ can be attributed mainly to two reasons. Firstly, 69.4% of patients who underwent MMOD and 96.7% of patients who underwent OFA were transferred from the OR to the ICU in SpVent, compared to 22.4% in REMI. This results in a shorter duration of IMV_ICU_. Secondly, the reduced need for vasopressor support in the ICU may have also resulted in a shorter LOS in the ICU. Although the MMOD group has better outcomes than REMI, only the OFA strategy proved to be independently associated with the considered endpoints, being the greater contributor to MMOD results.

### 4.1. Ventilation and Airway Patency

The prolonged duration of IMV is a well-recognised risk factor for respiratory tract infections, which is in agreement with our data. Anaesthetic management and decision-making regarding weaning from the ventilator in the OR significantly affects IMV_ICU_ duration [8,26]. Opioids are potent depressors of the respiratory drive and remifentanil is a short-acting and potent opioid that should be substituted for a longer-acting opioid at the end of surgery in order to avoid acute withdrawal syndrome. Prolonged administration of high doses of remifentanil aggravates the need for larger amounts of longer-acting opioids [11,12,13]. Fentanyl perfusion is often the first-line drug choice in the ICU. Both REMI and continuous postoperative fentanyl infusion for analgesia resulted in longer IMV_ICU_ duration, increased incidence of pneumonia, and a longer time required to start tracheal cannula occlusion training and cannula removal.

An excessive amount of tracheal secretions was frequently described in our cohort, even in patients without signs of respiratory infection. This observation can be attributed to intraoperative and IMV_ICU_, surgical aggression to the neck, presence of blood in the pharynx, and relative immobility [7]. Secretions also represent a source of bacterial contamination for cervical tissues. Competent and continuous evaluation of the cannula’s patency, the patient’s ability to manage the secretions, and suction when needed are mandatory. Two severe complications in our cohort were related to postoperative airway obstruction resulting in cardiac arrest. In both cases, the patients were submitted to an emergency tracheostomy, with one dying within 30 days and the other recovering without sequelae.

### 4.2. Pain and Adverse Effects from Analgesics

The incidence of moderate or severe postoperative pain and analgesic-attributable adverse effects was reduced in MMOD and OFA strategies. The strong correlation between the MMOD and multimodal postoperative analgesic strategies using PCA-KM, which were associated with better outcomes in pain, impedes an adequate separate analysis. The use of a multimodal strategy is known to decrease pain, improve satisfaction, and reduce opioid use and its adverse effects [11,12]. The most common side effects were obstipation, the delayed function of the GI tract, and delirium related to opioid abstinence. High-dose ketamine in the ICU (100–200 mg/kg) resulted in prolonged IMV_ICU_ and ICU_LOS_.

### 4.3. Blood Loss and Transfusion

The average reported blood loss in our cohort was consistent with other published literature. Blood loss is frequently associated with flap failure; however, our data do not demonstrate a significant association. What is worrisome is that estimated blood loss via HbCalc (derived from preoperative and 12 h postoperative Hb concentration) is significantly higher than the anaesthesiologists’ estimation (Total +906 mL, REMI +1010 mL, MMOD +738 mL, OFA +781 mL). Recent articles on other types of surgery trying to precisely measure blood loss indicate that blood losses are probably much higher than reported across the entire field [18], which is consistent with our observations.

The intraoperative administration of antiplatelet drugs resulted in reduced flap loss (7.7% vs. 10.3%) and overall complications (61.5% vs. 76.4%) when compared to non-administration; however, this was without statistical significance. Flap loss related to Tranexamic Acid (TNX) administration was lower when compared to non-administration (3.3% vs. 11.7% of patients, respectively) [27]. The same was observed with overall complications (63.3% vs. 74.0%).

Blood transfusion is traditionally associated with flap failure [6]; however, our data, just as other data published in the literature [28], show no significant association. Timely transfusions to reestablish circulating volume and content might be necessary during surgery and should not be withheld. Delayed transfusions and waiting for a fixed trigger value expose the patient to an adverse haemodynamic and perfusion status [23]. The authors propose a more dynamic approach, anticipating future losses and preferring to administer blood in the OR to assure circulatory stability and reduce the need for vasopressors in the ICU.

### 4.4. Fluid Administration and Fluid Losses

Prolonged surgery, involving significant blood loss and extensive fluid shifts, requires a carefully designed and calibrated fluid therapy plan. The REMI group administered significantly fewer fluids (8.50 mL/kg/h) than the MMOD group (9.96 mL/kg/h) and OFA subgroup (10.49 mL/kg/h). In our study, the rates are slightly higher than those published. Our calculations for intraoperative time did not include anaesthetic induction or preparation (catheter placement, difficult airway approach, transfer logistics), nor emergence from anaesthesia and exiting the OR. Those times were not reliable and were inconsistently recorded but typically amounted to more than 90 min.

The flap failure group showed greater fluid administration rates, in agreement with the literature, but without a statistically significant association. Conversely, our results show better performance of an increased fluid rate that cannot be dissociated from the MMOD and OFA strategies. We cannot exclude its impact on the overall strategy but neither can we affirm its independent effect detached from the other components.

Calculating “on-the-fly” fluid losses to evaporation, transudation to surgical gauzes and tissues, and oedema formed in the soft tissues reliably and with high precision is impossible [9]. Estimates must be made and different formulas and strategies exist that are operator-dependent. REMI significantly reported lower “hidden” losses (REMI 2.53 mL/kg/h; MMOD 4.66 mL/kg/h; OFA 6.38 mL/kg/h), including 24 (41%) patients with zero mL of insensitive losses recorded. In a 10-hour major surgery, though not recorded, they clearly were accounted for when making fluid administration decisions, as shown by REMI’s higher NFB of +2228 mL in comparison with MMOD’s +1311 mL and OFA’s +344 mL. When accounting for blood losses estimated from HbCalc, the intraoperative NFB remains positive but below +1000 mL, which is within the ideal target suggested by the RELIEF trial for fluid management in major surgery [29].

The underestimation of haemorrhage and the disconsideration of other fluid loss, and its propagation in NFB calculations, deteriorates the ability to make purposeful decisions regarding fluid administration. This results in marked discrepancies in fluid therapy, even for equivalent NFB targets. Nevertheless, none of the fluid-related variables independently affect the outcomes in our cohort.

Automatic software calculations of NFB ignoring those losses could have obfuscated the clinical evaluation, resulting in fluid administration deficits. The misleading information propagated to the ICU and concurred with the use of postoperative vasopressors, which were associated with flap necrosis.

### 4.5. Sympathomimetic Drugs

The myth that using inotropic and vasoactive drugs during the intraoperative period negatively impacts reconstruction outcomes has been disproven by several authors [7,23,24,30]. Nevertheless, aminergic support could be interpreted as an advanced marker for extensive blood and fluid loss resulting in acute hypovolemia. Judicious use to maintain perfusion and haemodynamic stability while measures are taken to reestablish normovolaemia, such as correcting anaemia, oncotic pressure, and coagulation factors, if needed, is key to achieving better outcomes [2,23,24,24]. The authors believe that selecting inotropes, such as dobutamine—in detriment of vasoconstrictors—after the anastomosis is completed, with a target increase of 10–15% of basal arterial pressure, yields immediate improvement of flap reperfusion, based on the intraoperative visual analysis reported by the surgical team. The vasodilation properties of dobutamine on flap arteries are unopposed by nerve-mediated compensatory vasoconstriction and might be beneficial. Conversely, systemic vasodilation, particularly at low rates, in a hypovolaemic patient results in hypotension. Steps must be taken to ensure adequate volaemia when the time comes to initiate “controlled hypertension” with dobutamine.

### 4.6. Controlled Hypotension, MMOD, and OFA

The excision of neoplastic or necrotic tissue, neck dissection, and flap harvesting are prone to major blood losses and benefit from controlled hypotension [31]. The REMI group achieved this goal by increasing remifentanil infusion rates. The MMOD group adopted two different strategies: (1) starting a low-rate remifentanil infusion for this sole purpose while assuring analgesia was previously established in a multimodal non-opioid manner; (2) administering the nitric-oxide-mediated vasodilation, recurring mainly to sodium nitroprusside, that originated the OFA subgroup. The administration of a vasodilator in the OR is not associated with better outcomes after accounting for the anaesthetic strategy. Haemodynamic results from direct vasodilation, without a remifentanil-mediated negative inotropic effect, were used to subjectively infer volaemic status and guide fluid therapy decisions.

### 4.7. AAS, Heparin, and TNX

The MMOD and OFA strategies include the administration of an anti-fibrinolytic agent (15 mg/kg bolus of tranexamic acid) at the start of surgery [27,32], aiming to reduce haemorrhage during tissue extirpation. When anastomoses are completed, an intravenous bolus of an antiplatelet agent (900 mg of lysin acetylsalicylate) is administered, aiming to reduce the formation of arterial thrombi due to the turbulent flow and endothelial trauma. Both drugs were associated with improved results but only achieved statistical significance when decoupled from the anaesthetic strategy. Heparin was administered in some patients, only in the REMI group, and was associated with increased flap failure and overall complications (*p* < 0.01).

### 4.8. Comorbidities

Many studies have evaluated the correlation between medical comorbidities and the occurrence of both surgical and medical perioperative complications. Other authors’ reported incidences of disease varied between series; our data shows incidence rates within those ranges [20,32].

Our results show, contrary to some of the published literature [20,21], that neither age nor other comorbidities identified in the population had a significant impact on surgical outcomes for reconstruction.

When analysing follow-ups, it was found that 14 (13.1%) patients were dead within one year (date of death 120 ± 83 days after surgery, including postoperative hospitalization deaths). None of those patients had a flap loss but 85.7% had some type of complication during the hospitalization. Half of them suffered from postoperative pneumonia. Six of them were in the MMOD group. The duration of hospitalization was greater (37.8 ± 18.3 vs. 24.0 ± 14.4 days) and the patients were about 10 years older at the time of surgery (68.8 ± 12.0 vs. 58.5 ± 14.3). These data show agealthough not directly associated with flap loss [20], it clearly has in impact in the long-term survival and should be considered when deciding whether to intervene in cases of older and more fragile patients, particularly when the reason for surgery is radionecrosis.

### 4.9. Paradigm Shift in Surgical-Related Complications

Routine and experienced teams, acquainted with working together, benefit from increased awareness and better verbal and nonverbal communication. Coordination between the surgical and anaesthetic personnel is essential to assure multidisciplinary decision-making, predict surgical events, and anticipate decisions in a timely manner.

Timing the controlled hypotension to avoid blood losses during dissections, and the relative hypertension during surgery to improve flap perfusion and oxygenation, requires coordination between the surgical and anaesthetic teams. Mimicking the postoperative haemodynamic status, predictably with periods of increased arterial blood pressure, is essential to allow competent surgical haemostasis and directly impacts the risk of haematoma formation [33]. Adequate tissue perfusion and oxygenation—and implicitly volaemic status and Hb concentration—in the postoperative period is critical to reduce flap infections and other flap complications.

The decision to intervene regarding the administration and timing of fluids, transfusions, hypothermia prevention, and the use of other drugs (e.g., sympathomimetics, antifibrinolytics, antiplatelets, anticoagulants, analgesics, etc.) are mainly at the discretion of the anaesthesiologist. Those decisions are dependent on the strategy chosen (REMI, MMOD, OFA) and are simultaneously conditioned by it. Our clinical experience, and the results from this study and others [33,34], indicate there is an association between anaesthetic conduct and complications traditionally attributed only to surgical factors, such as haematoma formation, surgical site infections, microvascular thrombosis, and anastomosis patency. It is the rationale for the inclusion of these endpoints in overall complications, instead of isolating them as surgically driven. The reverse is also true, considering that more than 25% of the variation in risk-adjusted complication rates—surgical and medical—can be attributable to the surgeon’s technical skills [35]. Since our cohort is from a single centre with a consistent surgical team, inter-surgeon variability is mitigated.

### 4.10. Limitations

Our study has many limitations. Our analysis was limited by software restrictions and a lack of connections to the automated infusers, which ignored various adjustments to flow rates and total doses of remifentanil, propofol and vasopressors. The same applies to the total doses of analgesia administered in the PSW and to variable rates of perfusions in the ICU.

Details about the clinical evolution during hospitalisation were dependent on the availability and quality of the medical records.

The lack of institutional protocols regarding fluid administration, blood transfusion, and the selection of sympathomimetic drugs (timing, type, rate, and goals) also influenced our results. Undoubtedly, varied criteria were used; this is reflected in the differences encountered between anaesthetists.

The aim of our study was to determine whether patient characteristics and anaesthetic strategy were associated with complications and failure rates; however, our study design cannot attribute or differentiate causality since the surgical technique was assumed to be uniform and only rarely a surgical cause, such as when an anastomosis leak, insufficient haemostasis, or drain malposition could be clearly identified.

## 5. Conclusions

Multimodal, and especially opioid-free, anaesthetic strategies provided better outcomes than opioid-based regimens. Flap failure and overall complications were reduced with MMOD and OFA protocols. Pneumonia incidence and other medical complications were significantly reduced in the OFA group. Those results are probably related to the reduced IMV_ICU_ duration and less need for ICU vasopressor use in the OFA group. OFA also shortened the ICU_LOS_, DoH, and its associated costs.

A slightly positive intraoperative NFB, with meticulous consideration of insensitive and “hidden” fluid losses, as well as timely blood transfusions, provided better results. Intraoperative haemodynamic manipulation with sympathomimetics was preferred in the OFA group and did not compromise any endpoint. The need for vasopressors in the ICU is associated with flap necrosis.

The administration of tranexamic acid in the early intraoperative period, as well as the administration of antiplatelet agents after the anastomoses, is a cornerstone of MMOD and OFA. It did not increase thromboembolic events, nor affect blood loss or haematoma formation, but is a potential contributor to the improved outcomes in OFA.

Multimodal intraoperative and postoperative analgesia, used in MMOD and OFA, improved pain scores and reduced ARC.

We cannot isolate the effect of each component of the MMOD and OFA strategies regarding its success. We hypothesise that the strategy as a whole is responsible for the positive results observed, as opposed to any individual intervention. Prospective studies isolating each component are necessary to develop recommendations and improve the standardisation of care.

## Figures and Tables

**Table 1:** Surgical Technique details and Surgical Outcomes.

N		Integrated		Small Defects		Dehiscence		Partial Necrosis		Loss of Flap	
**Donor Site: AL Thigh**	**15**	**8**	**(53.3%)**			**3**	**(20%)**	**3**	**(20%)**	**1**	**(6.7%)**
**Cancer Extirpation**	15	8	(53.3%)			3	(20%)	3	(20%)	1	(6.7%)
**Neck Dissection**	15	8	(53.3%)			3	(20%)	3	(20%)	1	(6.7%)
**Prior Surgery**	4					1	(25%)	2	(50%)		
**Radiation Exposure**	3					1	(33.3%)	1	(33.3%)		
**Donor Site: Forearm**	**26**	**19**	**(73.1%)**	**2**	**(7.7%)**			**4**	**(15.4%)**	**1**	**(3.8%)**
**Cancer Extirpation**	26	19	(73.1%)	2	(7.7%)			4	(15.4%)	1	(3.8%)
**Neck Dissection**	19	13	(68.4%)	2	(10.5%)			3	(15.8%)	1	(5.3%)
**Prior Surgery**	11	6	(54.5%)					2	(18.2%)	1	(9.1%)
**Radiation Exposure**	2	2	(100%)								
**Donor Site: Iliac Crest**	**17**	**10**	**(58.8%)**	**2**	**(11.8%)**	**2**	**(11.8%)**	**2**	**(11.8%)**	**1**	**(5.9%)**
**Cancer Extirpation**	14	10	(71.4%)	1	(7.1%)	2	(14.3%)			1	(7.1%)
**Neck Dissection**	5	4	(80%)			1	(20%)				
**Prior Surgery**	6	1	(16.7%)			2	(33.3%)	2	(33.3%)	1	(16.7%)
**Radiation Exposure**	4					1	(25%)	2	(50%)	1	(25%)
**Donor Site: Fibula**	**39**	**11**	**(28.2%)**	**14**	**(35.9%)**	**6**	**(15.4%)**	**3**	**(7.7%)**	**5**	**(12.8%)**
**Cancer Extirpation**	20	7	(35%)	5	(25%)	6	(30%)	2	(10%)		
**Neck Dissection**	11	4	(36.4%)	2	(18.2%)	3	(27.3%)	2	(18.2%)		
**Prior Surgery**	25	6	(24%)	9	(36%)	3	(12%)	2	(8%)	5	(20%)
**Radiation Exposure**	21	7	(33.3%)	6	(28.6%)	1	(4.8%)	2	(9.5%)	5	(23.8%)
**Donor Site: Other**	**10**	**5**	**(50%)**	**2**	**(20%)**	**1**	**(10%)**			**2**	**(20%)**
**Cancer Extirpation**	8	4	(50%)	2	(25%)	1	(12.5%)			1	(12.5%)
**Neck Dissection**	6	3	(50%)	1	(16.7%)	1	(16.7%)			1	(16.7%)
**Prior Surgery**	3	2	(66.7%)							1	(33.3%)
**Radiation Exposure**	2	1	(50%)							1	(50%)
**TOTAL**	107	53	49.5%	20	18.7%	12	11.2%	12	11.2%	10	9.3%

Outcomes grouped by donor-site using Clavien-Dindo Classification System. Integrated: Clavien-Dindo = 0, Small Defects = 1 (Any deviation from the normal postoperative course without the need for pharmacological treatment or surgical, endoscopic and radiological interventions), Dehiscence = 2 (Requiring pharmacological treatment with drugs other than such allowed for grade I complications), Partial Necrosis = 3 (Requiring surgical, endoscopic or radiological intervention), Loss of Flap = 4 (Life-threatening complication [including CNS complications] ∗ requiring IC/ICU-management). Class 5 (Death of a patient), is considered elsewhere. AL Thigh: Antero Lateral Thigh. No statistically significant association between FTR donor site and measured outcomes.

**Table 2:** Logistic Regression.

Excluded Variables	Included in the Equation
**Overall** **complications**	Comorbidities*, Sex (cat), Age, BMI, ASA ≥ 3 Sympathomimetics in the OR OFA (OR 0.30, CI 95% 0.04–0.89, *p* = 0.011) (cat), Fluid administered (mL), Blood loss reported (mL), Transfusion in Radionecrosis (OR 5.3, CI 95% 1.12–25.41, *p* = 0.035) OR (cat), RBC transfused in OR (N Units), FTT Donor-Site
**Flap loss**	OFA, Comorbidities*, Sex (cat), Age, BMI, Sympathomimetics in the OR BMI (OR 0.76, CI 95%, 0.582–0.995) (cat), Fluid administered (mL), Blood loss reported (mL), Transfusion in Radionecrosis (OR 4.63, CI 95% 0.97–21.95) OR (cat), RBC transfused in OR (N Units), FTT Donor-Site ASA status ≥ 3 (OR 11.77, CI 95% 1.76–78.82)
*Comorbidities	Preoperative INR value, Preoperative platelet count, and Preoperative Hb concentration, as continuous variables. Binomial categories were considered for: Hypertension, Cardiac Insufficiency, Vascular Disease, Diabetes Mellitus type 2, Hiperlipidaemia, Respiratory Disease, History of different neoplasm, Chronic Pain, Depression, Alcohol consumption, Tobacco use, Anticoagulation therapy, Sedative use, and Opioid use.

Characterization of the binary logistic regression performed for Overall Complications and Flap Loss. Excluded variables in both models included all comorbidities that are listed as binary categories. BMI: Body Mass Index, FTT: Free Tissue Transfer, RBC: Red Blood Cells. Results are presented using odds ratio and 95% confidence intervals.

**Table 3:** Summary of Surgical Procedure Details (Surgical Indication, Donor site, Length of Surgery, Outcomes) and Anaesthetic Strategy.

	N	Complications		Flap Loss		Surgery Duration		REMI		MMOD		OFA	
**Oral Cancer**	**48**	**33**	**(68.8%)**	**3**	**(6.3%)**	**9H 45′**	**(585′)**	**26**	**(54.2%)**	**22**	**(45.8%)**	**16**	**(33.3%)**
Anterolateral Thigh	13	9	(69.2%)	1	(7.7%)	9H 52′	(592′)	10	(76.9%)	3	(23.1%)	2	(15.4%)
Forearm	20	11	(55%)	1	(5%)	9H 10′	(550′)	12	(60%)	8	(40%)	4	(20%)
Iliac Crest	1	0	(0%)	0	(0%)	7H 45′	(465′)	0	(0%)	1	(100%)	1	(100%)
Fibula	9	8	(88.9%)	0	(0%)	11H 12′	(672′)	3	(33.3%)	6	(66.7%)	6	(66.7%)
Other	5	5	(100%)	1	(20%)	9H 32′	(572′)	1	(20%)	4	(80%)	3	(60%)
**RadioNecrose**	**20**	* 18	**(90%)**	* 6	**(30%)**	**9H 39′**	**(579′)**	**11**	**(55%)**	**9**	**(45%)**	**5**	**(25%)**
Iliac Crest	3	3	(100%)	0	(0%)	8H 21′	(501′)	1	(33.3%)	2	(66.7%)	1	(33.3%)
Fibula	16	14	(87.5%)	5	(31.3%)	9H 56′	(596′)	9	(56.3%)	7	(43.8%)	4	(25%)
Other	1	1	(100%)	1	(100%)	9H 7′	(547′)	1	(100%)	0	(0%)	0	(0%)
**Face-Skin Cancer**	**9**	**4**	**(44.4%)**	**0**	**(0%)**	**7H 35′**	**(455′)**	**6**	**(66.7%)**	**3**	**(33.3%)**	**3**	**(33.3%)**
Anterolateral Thigh	1	0	(0%)	0	(0%)	8H 3′	(483′)	0	(0%)	1	(100%)	1	(100%)
Antebraquial	6	3	(50%)	0	(0%)	7H 25′	(445′)	5	(83.3%)	1	(16.7%)	1	(16.7%)
Other	2	1	(50%)	0	(0%)	7H 51′	(471′)	1	(50%)	1	(50%)	1	(50%)
**Maxilar Cancer**	**13**	**8**	**(61.5%)**	**0**	**(0%)**	**9H 50′**	**(590′)**	**5**	**(38.5%)**	**8**	**(61.5%)**	**4**	**(30.8%)**
Anterolateral Thigh	1	0	(0%)	0	(0%)	8H 20′	(500′)	0	(0%)	1	(100%)	1	(100%)
Iliac Crest	5	4	(80%)	0	(0%)	10H 18′	(618′)	2	(40%)	3	(60%)	2	(40%)
Fibula	7	4	(57.1%)	0	(0%)	9H 44′	(584′)	3	(42.9%)	4	(57.1%)	1	(14.3%)
**Mandible Cancer**	**12**	**8**	**(66.7%)**	**1**	**(8.3%)**	**8H 56′**	**(536′)**	**8**	**(66.7%)**	**4**	**(33.3%)**	**1**	**(8.3%)**
Iliac Crest	8	5	(62.5%)	1	(12.5%)	8H 41′	(521′)	5	(62.5%)	3	(37.5%)	1	(12.5%)
Fibula	3	3	(100%)	0	(0%)	9H 46′	(586′)	2	(66.7%)	1	(33.3%)	0	(0%)
Other	1	0	(0%)	0	(0%)	8H 28′	(508′)	1	(100%)	0	(0%)	0	(0%)
**Trauma**	**5**	**5**	**(100%)**	**0**	**(0%)**	**9H 9′**	**(549′)**	**1**	**(20%)**	**4**	**(80%)**	**1**	**(20%)**
Fibula	4	4	(100%)	0	(0%)	9H 41′	(581′)	1	(25%)	3	(75%)	0	(0%)
Other	1	1	(100%)	0	(0%)	7H 0′	(420′)	0	(0%)	1	(100%)	1	(100%)
**TOTAL**	**107**	**76**	**(71%)**	**10%**	**(10%)**	**9H 26′**	**(566′)**	**57**	**(53.3%)**	**50**	**(46.7%)**	**30**	**(28%)**

* Significant association (*p* < 0.05) of radionecrosis with increased flap loss and overall complications. Reason for surgery ordered by frequency of the procedure in the cohort. There was no significant skewing in the distribution by type of procedure, nor reason for surgery between Anaesthetic Strategies. N: Number of Patients, REMI: Opioid-based anaesthetic strategy, MMOD: Multimodal Anaesthesia, OFA: Opioid-Free Anaesthesia and multimodal intraoperative analgesia.

**Table 4:** Patient Characteristics and Postoperative Outcomes.

	N	Flap Loss			Partial Necrosis		Surgical		Surgical-Site		Cervical		Pneumonia		Overall Complications			Death	
							Complications		Infection		Haematoma							within 1 Year	
**Age (Average)**	59.8 ± 14.4	56.2 ± 9.43 *		**(*p* = 0.088)**	63.8 ± 13.4		54.8 ± 13.8		59.3 ± 10.6		62.6 ± 15.0		60.2 ± 14.5		60.0 ± 13.9		(*p* = 0.781)	68.8 ± 12.0	
Phyisical Status			*	**(*p* = 0.044)**													(*p* = 0.084)		
ASA 1	6	0	(0%)		0	(0%)	0	(0%)	0	(0%)	0	(0%)	0	(0%)	2	(33.3%)		0	(0%)
ASA 2	55	2	(3.6%)		6	(10.9%)	16	(29.1%)	12	(21.8%)	13	(23.6%)	11	(20%)	42	(76.4%)		8	(14.5%)
ASA 3	46	8	(17.4%)		6	(13%)	10	(21.7%)	12	(26.1%)	10	(21.7%)	12	(26.1%)	32	(69.6%)		6	(13%)
BMI < 18	12	3	(25%) *	**(*p* = 0.048)**	3	(25%)	7	(58.3%)	3	(25%)	6	(50%)	4	(33.3%)	9	(75%)	(*p* = 0.748)	4	(33.3%)
BMI > 25	50	2	(4%)	(*p* = 0.075)	5	(10%)	10	(20%)	10	(20%)	10	(20%)	9	(18%)	33	(66%)	(*p* = 0.283)	6	(12%)
Normal BMI	45	5	(11.1%)		4	(8.9%)	9	(20%)	11	(24.4%)	8	(17.8%)	10	(22.2%)	34	(75.6%)		4	(8.9%)
**Previous Comorbidities**																			
Arterial Hipertension	39	2	(5.1%)	(*p* = 0.256)	6	(15.4%)	7	(17.9%)	8	(20.5%)	10	(25.6%)	9	(23.1%)	27	(69.2%)	(*p* = 0.756)	6	(15.4%)
Cardiac Insufficiency	8	1	(12.5%)	(*p* = 0.750)	2	(25%)	4	(50%)	1	(12.5%)	5	(62.5%)	3	(37.5%)	4	(50%)	(*p* = 0.173)	3	(37.5%)
Vascular Events	12	0	(0%)	(*p* = 0.238)	1	(8.3%)	4	(33.3%)	2	(16.7%)	3	(25%)	3	(25%)	8	(66.7%)	(*p* = 0.724)	3	(25%)
Diabetes Mellitus	11	1	(9.1%)	(*p* = 0.976)	2	(18.2%)	1	(9.1%)	4	(36.4%)	3	(27.3%)	2	(18.2%)	9	(81.8%)	(*p* = 0.405)	1	(9.1%)
Dyslipidemia	40	2	(5%)	(*p* = 0.233)	4	(10%)	5	(12.5%)	10	(25%)	9	(22.5%)	**4**	(10%) *	27	(67.5%)	(*p* = 0.534)	4	(10%)
Respiratory Disease	17	2	(11.8%)	(*p* = 0.709)	3	(17.6%)	5	(29.4%)	6	(35.3%)	5	(29.4%)	5	(29.4%)	12	(70.6%)	(*p* = 0.965)	4	(23.5%)
Other Neoplasy	15	3	(20%)	(*p* = 0.126)	2	(13.3%)	2	(13.3%)	5	(33.3%)	3	(20%)	6	(40%)	13	(86.7%)	(*p* = 0.150)	2	(13.3%)
Chronic Pain	25	4	(16%)	(*p* = 0.192)	4	(16%)	10	(40%)	8	(32%)	8	(32%)	6	(24%)	18	(72%)	(*p* = 0.903)	5	(20%)
**Consumption Habits**																			
Alcohol use	29	4	(13.8%)	(*p* = 0.443)	3	(10.3%)	9	(31%)	8	(27.6%)	6	(20.7%)	6	(20.7%)	20	(69%)	(*p* = 0.354)	4	(13.8%)
Alcohol abuse	15	2	(13.3%)		3	(20%)	5	(33.3%)	6	(40%)	5	(33.3%)	6	(40%)	13	(86.7%)		1	(6.7%)
Tobbaco Use	29	4	(13.8%)	(*p* = 0.346)	3	(10.3%)	9	(31%)	8	(27.6%)	3	(10.3%)	9	(31%)	22	(75.9%)	(*p* = 0.721)	3	(10.3%)
Ex-Smoker	14	0	(0%)		2	(14.3%)	4	(28.6%)	2	(14.3%)	5	(35.7%)	3	(21.4%)	9	(64.3%)		2	(14.3%)
**Surgical-Site History**																			
Tumor Recidive	28	2	(7.1%)	(*p* = 0.344)	4	(14.3%)	7	(25%)	8	(28.6%)	7	(25%)	8	(28.6%)	19	(67.9%)	(*p* = 0.814)	4	(14.3%)
Previous Surgery	49	8	(16.3%)	(*p* = 0.137)	8	(16.3%)	14	(28.6%)	14	(28.6%)	10	(20.4%)	15	(30.6%)	37	(75.5%)	(*p* = 0.752)	5	(10.2%)
Radiotherapy History	32	7	(21.9%)	(*p* = 0.063)	5	(15.6%)	10	(31.3%)	9	(28.1%)	8	(25%)	10	(31.3%)	26	(81.3%)	(*p* = 0.415)	4	(12.5%)
**Medication History**																			
Antiplatelet	7	**1**	(14.3%) *	**(*p* = 0.019)**	2	(28.6%)	2	(28.6%)	2	(28.6%)	3	(42.9%)	3	(42.9%)	7	(100%)	(*p* = 0.376)	3	(42.9%)
Anticoagulation	4	0	(0%)	(*p* = 0.513)	0	(0%)	1	(25%)	1	(25%)	2	(50%)	0	(0%)	2	(50%)	(*p* = 0.345)	1	(25%)
Sedatives	31	2	(6.5%)	(*p* = 0.511)	1	(3.2%)	9	(29%)	4	(12.9%)	9	(29%)	5	(16.1%)	21	(67.7%)	(*p* = 0.645)	5	(16.1%)
Opioids	15	2	(13.3%)	(*p* = 0.562)	3	(20%)	4	(26.7%)	6	(40%)	6	(40%)	4	(26.7%)	11	(73.3%)	(*p* = 0.832)	4	(26.7%)
**Total**	**107**	**10**	**(9.3%)**		**12**	**(11.2%)**	**26**	**(24.3%)**	**24**	**(22.4%)**	**24**	**(22.4%)**	**23**	**(21.5%)**	**76**	**(71%)**		**14**	**(13.1%)**

* Statistically significant association (*p* < 0.05) with the outcome. *p*-values from T-test for continuous variables, Mann-Whitney U test for non-normal continuous, and Chi-squared or Fisher’s exact test for categorical variables, when aplicable. Central tendency is presented in Mean ± SD. N: Number of patients, ASA: American Society of Anesthesiology, BMI: Calculated Body Mass Index, Partial Necrosis = Class 3 Clavien-Dindo (Requiring surgical, endoscopic or radiological intervention); Loss of Flap = Class 4 Clavien-Dindo (Life-threatening complication [including CNS complications] requiring IC/ICU-management).

**Table 5:** In-Hospital Mortality: Clinical Case Description.

In-Hospital Postoperative Death Description			
	Age	Reason for Surgery	Cause of Death
**Patient 1**	69	Oral tumour recidive after prior surgery and radiation therapy	Dislocation of gastrostomy tube with massive intraperitoneal haemorrhage and peritonitis. Bacteriemia with Enterobacter cloacae followed by sepsis and multiple organ failure.
**Patient 2**	50	Oral tumour recidive after prior surgery and radiation therapy	Nosocomial Pneumonia at the 4th postoperative day (Pseudomonas aeruginosa, MDR), not responding to large-spectrum antibiotics, with evolution to respiratory failure.
**Patient 3**	89	Tongue cancer	Postoperative airway obstruction, submitted to emergency cricothiroidostomy, resulting in diffuse anoxic encephalopathy and deterioration to neurological vegetative state.
**Patient 4**	80	Nasal septum carcinoma	Duodenal ulcera with massive digestive haemorrhage (hipovolaemic shock) + Fungemia + Urinary tract Infection and CVC contamination

**Table 6:** Fluid management and vasoactive drug administration.

	REMI		MMOD			OFA			Total	
**Blood Loss and IntrOp Transfusion**	58		49			30			107	
Reported Blood Loss (mL)	645 ± 368		845 ± 619		(*p* = 0.236)	827 ± 707		(*p* = 0.314)	739 ± 511	
Haemorrhage HbCalc (mL)	1581 ± 653		1669 ± 749		(*p* = 0.332)	1675 ± 897		(*p* = 0.918)	1664 ± 695	
Hb Decrease (g/dL)	4.61 ± 1.94		4.13 ± 1.96		(*p* = 0.986)	3.62 ± 2.18 *		**(*p* = 0.011)**	4.39 ± 1.95	
Transfusion IntraOp (N patients)	15 (25.9%)		22 (44.9%)		(*p* = 0.096)	16(53.3%)		(*p* = 0.079)	37 (34.6%)	
Units RBC IntraOp/Patient (N)	1.9 ± 1.2		2.8 ± 1.8 *		**(*p* = 0.004)**	3.4 ± 1.9 *		**(*p* = 0.001)**	2.43 ± 1.67	
**IntraOp Haemodinamic Interventions**										
Sympatomimetics use (N patients)	28 (48.3%)		24 (49.0%)			18 (60.0%)			52 (48.6%)	
Vasopressor IntraOp (N patients)	25 (43.1%)		15 (30.6%)			10 (33.3%)			40 (37.4%)	
Dobutamine IntraOp (N patients)	3 (5.2%)		9 (18.4%)			8 (26.7%)			12 (11.2%)	
Vasodilator IntraOp (N patients)	6 (10.3%)		19 (38.8%)			13 (43.3%)			25 (23.4%)	
**Fluid Therapy and Balance**										
Fluid IntraOp (mL/Kg)	90.6 ± 33.3		113 ± 34 *		**(*p* = 0.001)**	117 ± 36 *		**(*p* = 0.003)**	101 ± 35	
Fluid Rate IntraOp (mL/Kg/H)	8.59 ± 2.96		9.96 ± 2.25 *		**(*p* = 0.006)**	10.49 ± 2.32 *		**(*p* = 0.002)**	9.17 ± 2.74	
Diuresis IntraOp (mL/kg/h)	2.1 ± 1.36		3.1 ± 1.58 *		**(*p* = 0.004)**	3.23 ± 1.69 *		**(*p* = 0.002)**	2.56 ± 1.54	
Total Fluid Losses Reported (mL)	3907 ± 2448		6335 ± 3030 *		**(*p* = 0.002)**	7573 ± 2414 *		**(*p* = 0.001)**	5019 ± 2976	
“Hidden” Fluid Losses (mL/kg/h)	2.53 ± 2.69		4.66 ± 3.24 *		**(*p* = 0.001)**	6.38 ± 2.1 *		**(*p* = 0.001)**	3.38 ± 3.1	
Reported Net Fluid Balance (mL)	2228 ± 2018		1311 ± 2199 *		**(*p* = 0.027)**	344 ± 1751 *		**(*p* = 0.001)**	1808 ± 2142	
HbCalc Net Fluid Balance (mL)	1217 ± 2448		548 ± 2112		(*p* = 0.114)	−403 ± 1745 *		**(*p* = 0.001)**	911 ± 2180	
**Intensive Care Unit endpoints**										
Traqueostomy (N patients)	47 (81%)		40 (81.6%)			24 (80%)			87 (81.3%)	
IMV at ICU Admission	46 (79.3%)		15 (30.6%) *		**(*p* = 0.001)**	1 (3.3%) *		**(*p* = 0.001)**	61 (57%)	
T Mechanical Ventilation (H)	81 ± 97		33 ± 95		(*p* = 0.082)	9 ± 38 *		**(*p* = 0.029)**	82 ± 97	
Transfusion ICU (N)	22 (37.9%)		16 (32.7%)		(*p* = 0.411)	7 (23.3%)		(*p* = 0.068)	38 (35.5%)	
Units RBC ICU/Patient	2.23 ± 2.6		3 ± 1.9		(*p* = 0.133)	3 ± 2.2		(*p* = 0.241)	2.29 ± 2.3	
Vasopressor ICU (N)	27 (46.6%)		10 (20.4%) *		**(*p* = 0.005)**	3 (10%) *		**(*p* = 0.001)**	37 (34.6%)	

Analysis of end-results from clinical implementation of the anaesthetic protocol, showing differences regarding fluid and blood management and estimations, decision on vasoactive and inotrope use, and its consequences at ICU admission. Central tendency measures are presented in Mean ± SD. * Statistically significant (*p* < 0.05) variables that were independently correlated to the Anaesthetic Strategy. *p*-values under MMOD are MMOD vs. REMI. *p*-values under OFA are OFA vs. All Others. N: Number of patients; HbCalc: Calculated blood loss using Jaramillo et al. (2020) [18] method founded on the difference between preoperative haemoglobin concentration, postoperative nadir haemoglobin concentration, and volaemia calculations using Nadler’s formula; Hb: Haemoglobin concentration; IntraOp: Intraoperative period; RBC: Packed Red Blood Cells; IMV: Invasive Mechanical Ventilation; ICU: Intensive Care Unit; T: Time/Duration.

**Table 7:** Postoperative Respiratory-Related Outcomes and Anaesthetic Strategy. Respiratory-related outcomes grouped by Anaesthetic Strategy. Worth noting that even great changes in the outcome did not achieve statistical significance (e.g., MMOD has half the IMV_ICU_ duration from 66 to 33 hours, comparing to REMI). This is due to the reduced number of patients in the cohort, and influencing outliers (e.g., two patients with severe complications had more than 500H of IMV each).

	N	Spontaneous Vent.		IMV ICU	Start Training		Complete Clamp		Cannula Removal		Tracheal Secretions		Pneumonia		Complications	
		ICU Admission (N)		(Hours)	(Days)		(Days)		(Days)		(N)		(N)		Overall (N)	
**Opioid-Base**	**58**	**13**	**(22.4%)**	**66.3 ± 97.2**							**9**	**(15.5%)**	**16**	**(27.6%)**	**45**	**(77.6%)**
Traqueostomy	47	8	(17%)	75.7 ± 105	**13.3** ± 13.2		**18.9** ± 18.1		**21** ± 18.9		9	(19.1%)	14	(29.8%)	39	82.98%
Tracheal Tube	11	5	(45.5%)	27.3 ± 33							0	(0%)	2	(18.2%)	6	54.54%
**Multimodal**	**49**	**34**	(69.4%) *	**33.1 ± 94.7**	(*p* = 0.248)		(*p* = 0.131)		(*p* = 0.712)		**3**	(6.1%) *	**6**	(12.2%) *	**31**	(63.3%) *
Traqueostomy	40	26	(65%)	36.8 ± 103.2	**10.7** ± 5.4		**14.2** ± 5.9		**19.7** ± 10.6		3	(7.5%)	5	(12.5%)	28	70%
Tracheal Tube	9	8	(88.9%)	16.4 ± 39.3							0	(0%)	1	(11.1%)	3	33.33%
**Opioid-Free**	**30**	**29**	(96.7%) *	9.2 ± 38.4 *	(*p* = 0.550)		(*p* = 0.524)		(*p* = 0.842)		**1**	(3.3%) *	**4**	(13.3%) *	**16**	(53.3%) *
Traqueostomy	24	23	(95.8%)	11 ± 42.9	**11** ± 5.2		**15.1** ± 5.9		**19.9** ± 9.2		1	(4.2%)	3	(12.5%)	14	58.30%
Tracheal Tube	6	5	(80%)	2 ± 4.9							0	(0%)	1	(16.7%)	2	33.33%
**Total**	**107**	**47**	**(43.9%)**	**50.8 ± 97**							**11**	**(10.3%)**	**24**	**(22.4%)**	**76**	**(71%)**
Traqueostomy	87	34	(39.1%)	57.3 ± 105.2	**12.1** ± 10.4		**16.9** ± 14.1		**20.4** ± 15.6		11	(12.6%)	20	(23%)	67	77.01%
Tracheal Tube	20	13	(65%)	22.4 ± 35.9							0	(0%)	4	(20%)	9	45%

* Variables with significant statistical association (*p* < 0.05) with the respective outcome. Central tendency measures are presented in Mean ± SD. N: Number of patients; Spontaneous Vent. ICU Admission: Number of patients admitted in the Intensive Care Unit in Spontaneous Ventilation, regardless of airway device; IMV: Invasive Mechanical Ventilation; Start Training: Number of postoperative days to start tracheal cannula occlusion and training to breathe through cannula fenestration; Complete Clamp: Patient tolerates complete occlusion of fenestrated tracheal cannula; Cannula: Tracheostomy cannula.

**Table 8:** Intraoperative and Postoperative Drug Interventions and Pain-Related Outcomes. Characterization of the most relevant intraoperative drug usage and intraoperative analgesia management between REMI, OFA and MMOD and its pain-related outcomes. Is worth pointing the significant reduction of rocuronium consumption in the OFA group.

	REMI	MMOD	OFA	Total
	N = 58	N = 49	N = 30	N = 107
**Intraoperative Administration**				
Minor Analgesics (N)	37 (63.8%)	45 (91.8%)	29 (96.7%)	82 (76.6%)
Pre-Emptive Analgesia (N)	6 (10.3%)	31 (63.3%)	20 (66.7%)	37 (34.6%)
Ketamine Bolus (N)	15 (25.9%)	1 (2%)	0 (0%)	16 (15%)
Ketamine Infusion (N)	2 (3.4%)	48 (98%)	30 (100%)	50 (46.7%)
Lidocaine Bolus (N)	10 (17.2%)	1 (2%)	0 (0%)	11 (10.3%)
Lidocaine Perfusion (N)	17 (29.3%)	47 (95.9%)	30 (100%)	64 (59.8%)
MgSO4 Bolus (N)	10 (17.2%)	25 (51%)	9 (30%)	35 (32.7%)
MgSO4 Perfusion (N)	0 (0%)	14 (28.6%)	14 (46.7%)	14 (13.1%)
Rocuronium (IntraOp mg)	233 ± 113	250 ± 140	195 ± 85 *	240 ± 126
Rocuronium (mg/kg)	3.38 ± 1.63	3.75 ± 2.29	2.78 ± 1.14 *	3.55 ± 1.96
Rocuronium (mg/kg/h)	0.373 ± 0.17	0.379 ± 0.21	0.295 ± 0.12 *	0.376 ± 0.19
Long-Duration Opioid IntraOp (N)	28 (48.3%)	2 (4.1%)		30 (28%)
Morphine Equivalent (mg/Patient)	6.46 ± 3.48	3 ± 4.94		6.47 ± 3.48
Morphine Equivalent (mg/kg)	0.1 ± 0.06	0.04 ± 0.06		0.1 ± 0.06
**Safety and Efficiency**				
Analgesia Complications (N)	35 (60.3%)	22 (44.9%)	7 (23.3%) *	57 (53.3%)
Pain [VAS > 3] ICU (N)	9 (15.5%)	6 (12.2%)	2 (6.7%) *	15 (14%)
Pain [VAS > 3] PSW (N)	9 (15.5%)	4 (8.2%) *	2 (6.7%) *	13 (12.1%)

* Statistically significant (*p* < 0.05) association between the Anaesthetic Strategy and corresponding outcome. Central tendency measures are presented in Mean ± SD. MgSO_4_: Magnesium Sulphate; REMI: Opioid-based Anaesthesia group; MMOD: Multimodal Anaesthesia group; N: Number of patients; OFA: Opioid-Free Anaesthesia subgroup; PCA: Patient-Controlled Analgesia; PSW: Plastic Surgery Ward; VAS: Visual Analogue Scale; ICU: Intensive Care Unit.

**Table 9:** Postoperative Analgesic Interventions and Patient Outcomes. Six different types of postoperative analgesia strategies were identified. Worth noting that by transferring the patient to the ICU reduces the discretion of the anaesthesiologist in implementing the protocol of his choice. Complications related to analgesia included obstipation, prolonged digestive stasis, nausea and vomiting, and delirium.

	N	Pain ICU		Pain Ward		Complications		Flap Loss		Complications		IMV ICU	GI Nutrition	LOS ICU	Hospitalization
		(N)		(N)		Analgesia (N)		(N)		Overall (N)		(Hours)	(Hours)	(Days)	(Days)
**REMI**	**58**	**9**	**(15.5%)**	**9**	**(15.5%)**	**35**	**(60.3%)**	**7**	**(12.1%)**	**45**	**(77.6%)**	**66 ± 97**	**60 ± 35**	**5.3 ± 4.7**	**27.1 ± 17.0**
Fentanil (P)	14	2	(14.3%)	3	(21.4%)	12	(85.7%)	3	(21.4%)	12	(85.7%)	113	63	7.2	39.3
Tramadol (P)	30	2	(6.7%)	4	(13.3%)	15	(50%)	3	(10%)	22	(73.3%)	45	49	4.3	21.5
Ketamine (P) High-Dose	4	2	(50%)	1	(25%)	4	(100%)	0	(0%)	3	(75%)	108	111	6.8	41.5
Morphine (B)	5	1	(20%)	0	(0%)	3	(60%)	1	(20%)	3	(60%)	26	78	5	20
Minor Analgesics Only (UCI)	5	2	(40%)	1	(20%)	1	(20%)	0	(0%)	5	(100%)	67	58	5.4	22.4
**MMOD**	**49**	**6**	**(12.2%)**	**4**	(8.2%) *	**22**	**(44.9%)**	**3**	(6.1%) *	**31**	**(63.3%)**	**33 ± 95**	**46 ± 55**	3.9 ± 3.1 *	**24 ± 13.7**
Fentanil (P)	10	4	(40%)	1	(10%)	9	(90%)	2	(20%)	9	(90%)	112	78	5.1	30
Tramadol (P)	4	1	(25%)	0	(0%)	3	(75%)	0	(0%)	4	(100%)	42	48	7	28
Ketamine (P) High-Dose	1	0	(0%)	0	(0%)	1	(100%)	0	(0%)	1	(100%)	60	72	4	17
Morphine (B)	2	0	(0%)	1	(50%)	2	(100%)	0	(0%)	0	(0%)	8	30	3	10
PCA Ketamine	31	0	(0%)	1	(3.2%)	6	(19.4%)	1	(3.2%)	16	(51.6%)	8	37	3.3	23
Minor Analgesics Only (UCI)	1	1	(100%)	1	(100%)	1	(100%)	0	(0%)	1	(100%)	0	12	1	27
**OFA**	**30**	**2**	**(6.7%)**	**2**	(6.7%) *	**7**	(23.3%) *	**1**	(3.3%) *	**16**	(53.3%) *	9 ± 38 *	41 ± 56 *	3.4 ± 3.5 *	24.1 ± 13.1 *
Tramadol (P)	2	1	(50%)	0	(0%)	1	(50%)	0	(0%)	2	(100%)	30	60	7.5	34
PCA Ketamine	27	0	(0%)	1	(3.7%)	5	(18.5%)	1	(3.7%)	13	(48.1%)	8	40	3.2	23.3
Minor Analgesics Only (UCI)	1	1	(100%)	1	(100%)	1	(100%)	0	(0%)	1	(100%)	0	12	1	27
**Total**	**107**	**15**	**(14%)**	**13**	**(12.1%)**	**57**	**(53.3%)**	**10**	**(9.3%)**	**76**	**(71%)**	**51 ± 97**	**54 ± 45**	**4.7 ± 4.1**	**25.7 ± 5.6**
Fentanil (P)	24	6	(25%) *	4	(16.7%)	21	(87.5%) *	5	(20.8%)	21	(87.5%)	112 *	70	6.3 *	35.5 *
Tramadol (P)	34	3	(8.8%)	4	(11.8%)	18	(52.9%)	3	(8.8%)	26	(76.5%)	44	49	4.6	22.2
Ketamine (P) High-Dose	5	2	(40%) *	1	(20%) *	5	(100%) *	0	(0%)	4	(80%)	98 *	103 *	6.2	36.6 *
Morphine (B)	7	1	(14.3%)	1	(14.3%)	5	(71.4%)	1	(14.3%)	3	(42.9%)	21	64	4.4	17
PCA Ketamine	31	0	(0%)	1	(3.2%)	6	(19.4%)	1	(3.2%)	16	(51.6%)	8	37	3.3	22.6
Minor Analgesics Only (UCI)	6	3	(50%)	2	−33.30%	2	(33.3%)	0	(0%)	6	(100%)	56	50	4.7	23.2

* Statistically significant (*p* < 0.05) association between the Anaesthetic Strategy and corresponding outcome. Central tendency measures are presented in Mean ± SD for MMOD, REMI and OFA groups. For the sake of simplicity only the mean is presented for individual analgesia protocols. N: Number of patients; (P): Continuous Infusion/Perfusion; (B): Intermittent Boluses; ICU: Intensive Care Unit; GI Nutrition: Time from ICU Admission to Total Gastrointestinal/Enteral Nutrition; ICU: Intensive Care Unit; IMV: Invasive Mechanical Ventilation; LOS: length of stay; MMOD: Multimodal Anaesthesia group; OFA: Opioid-Free Anaesthesia group; REMI: Opioid-Base Anaesthesia group; PCA: patient-controlled analgesia; Minor Analgesics: Acetaminophen, Non-Steroid Anti-Inflammatory Drugs; Ketamine (P) High-Dose: Continuous infusion of ketamine 100–200 mg/h in the ICU.

**Table 10:** Postoperative Outcomes according to Anaesthetic Strategy and Surgical Indication. Analysis of the equivalent performance of the Anaesthetic Strategy in the different settings. Radionecrosis as surgical indication is an independent factor for worse outcomes. The reduced number of patients in the cohort and influencing outliers (e.g., OFA barely achieved significant association DoH [*p* = 0.048], despite almost 5 days mean reduction in hospitalization; the topmost five patients had DoH averaging 77 days).

	N	Overall		Surgical		Flap Loss		Hematoma		Site Infection		Pneumonia		Secretions		ICU Lenght of Stay	Hospitalization
		Complications		Complications												(Days)	(Days)
**REMI**	**58**	**45**	**(77.6%)**	**13**	**(22.4%)**	**7**	**(12.1%)**	**13**	**(22.4%)**	**15**	**(25.9%)**	**17**	**(29.3%)**	**8**	**(13.8%)**	**4.84 ± 4.74**	**29.11 ± 17.02**
Head & Neck Cancer	45	33	(73.3%)	8	(17.8%)	10	(22.2%)	11	(24.4%)	12	(26.7%)	10	(22.2%)	7	(15.6%)	5.67 ± 5.22	26.3 ± 16.95
Radionecrosis	12	10	(83.3%)	5	(41.7%)	4	(33.3%)	2	(16.7%)	3	(25%)	2	(16.7%)	1	(8.3%)	4.27 ± 2.28	30.09 ± 19.17
Other Causes	2	2	(100%)	0	(0%)	0	(0%)	0	(0%)	0	(0%)	0	(0%)	0	(0%)	3 ± 0	28.5 ± 7.78
**MMOD**	**49**	**31**	**(63.3%)**	**13**	**(26.5%)**	**3**	**(6.1%)**	**11**	**(22.4%)**	**9**	**(18.4%)**	**7**	(14.3%) *	**3**	**(6.1%)**	3.92 ± 3.06 *	**24 ± 13.66**
Head & Neck Cancer	36	20	(55.6%)	8	(22.2%)	1	(2.8%)	9	(25%)	6	(16.7%)	4	(11.1%)	1	(2.8%)	4.05 ± 3.23	22.02 ± 12.34
Radionecrosis	10	9	(90%)	5	(50%)	2	(20%)	2	(20%)	2	(20%)	4	(40%)	1	(10%)	3.6 ± 2.63	33.3 ± 15.59
Other Causes	3	3	(100%)	0	(0%)	0	(0%)	0	(0%)	1	(33.3%)	0	(0%)	1	(33.3%)	3 ± 2	22.33 ± 14.74
**OFA**	**30**	**16**	(53.3%) *	**7**	**(23.3%)**	**1**	(3.3%) *	**8**	**(26.7%)**	**4**	**(13.3%)**	**4**	(13.3%) *	**1**	(3.3%) *	3.43 ± 3.51 *	24.1 ± 13.1 *
Head & Neck Cancer	24	11	(45.8%)	5	(20.8%)	1	(4.2%)	7	(29.2%)	3	(12.5%)	3	(12.5%)	1	(4.2%)	3.91 ± 3.77	22.96 ± 11.04
Radionecrosis	6	5	(83.3%)	2	(33.3%)	0	(0%)	1	(16.7%)	1	(16.7%)	2	(33.3%)	0	(0%)	1.83 ± 0.75	33 ± 18.24
**TOTAL**	**107**	**76**	**(71%)**	**26**	**(24.3%)**	**10**	**(9.3%)**	**24**	**(22.4%)**	**24**	**(22.4%)**	**24**	**(22.4%)**	**11**	**(10.3%)**	**4.67 ± 4.1**	**25.7 ± 15.57**
Head & Neck Cancer	82	53	(64.6%)	16	(19.5%)	4	(4.9%)	20	(24.4%)	18	(22%)	19	(23.2%)	8	(9.8%)	4.93 ± 4.48	24.39 ± 15.11
Radionecrosis	21	19	(90.5%) *	10	(47.6%) *	6	(28.6%) *	4	(19%)	5	(23.8%)	6	(28.6%)	2	(9.5%)	3.95 ± 2.42	31.61 ± 17.2 *
Other Causes	5	5	(100%)	0	(0%)	0	(0%)	0	(0%)	1	(20%)	0	(0%)	1	(20%)	3 ± 1.15	24.8 ± 11.63

* Statistically significant (*p* < 0.05) association between the Anaesthetic Strategy and corresponding outcome. Central tendency measures are presented in Mean ± SD. N: Number of patients, Surgical Complications: Identifiable surgical complications (e.g., Anastomosis leak, Vascular kinking, Drain malposition or obstruction, Defective Haemostasis); Secretions: Excessive tracheal secretions reported needing regular suction; ICU: Intensive Care Unit; Hospitalization: Length of stay (LOS) in the hospital after surgery until administrative discharge, including time in intermediate care, long-term care, and delays due to non-clinical motives (e.g., Lack of social support at home, poor economic conditions, administrative delays—all are common in Portuguese hospitals and increase LOS).

**Table 11:** Intraoperative administration of anticoagulants, antiplatelet and antifibrinolytic agents and hemorrhage-related outcomes.

		N	Blood Loss	Blood Loss	Hb Decrease	Transfusion	Transfusion		Vasopressor ICU		Hematoma		Flap Loss		Complications		Complications	
			Reported (mL)	Calculated (mL)	(g/dL)	(Units RBC)	N		N		N		N		Surgical (N)		Overall (N)	
**Heparin**		**11**	**1345** ± 838	**1884** ± 908	**5.12** ± 1.97	**1.72** ± 2.57	**5**	**(45.5%)**	**8**	**(72.7%)**	**6**	**(54.5%)**	**4**	**(36.4%)**	**7**	**(63.6%)**	**9**	**(81.8%)**
Remifentanil	(13.8%)	8	731	1603	4.43	0.75	2	(25%)	5	(62.5%)	4	(50%)	3	(37.5%)	4	(50%)	6	(75%)
Multimodal	(6.1%)	3	1900	2635	7	4.33	2	(66.7%)	3	(100%)	2	(66.7%)	1	(33.3%)	3	(100%)	3	(100%)
Opioid-Free	(3.3%)	1	3250	4257	9.7	6	1	(100%)	1	(100%)	1	(100%)	0	(0%)	1	(100%)	1	(100%)
**AAS + TNX**		**23**	**853** ± 463	**1510** ± 520	**3.82** ± 1.66	**0.61** ± 1.34	**7**	**(30.4%)**	**4**	**(17.4%)**	**3**	**(13%)**	**1**	**(4.3%)**	**5**	**(21.7%)**	**15**	**(65.2%)**
Remifentanil	(3.4%)	2	950	1083	3.35	0.61	0	(0%)	0	(0%)	0	(0%)	0	(0%)	0	(0%)	1	(50%)
Multimodal	(42.9%)	21	844	1551	3.86	0.67	7	(33.3%)	4	(19%)	3	(14.3%)	1	(4.8%)	5	(23.8%)	14	(66.7%)
Opioid-Free	(46.7%)	14	830	1500	3.18	0.71	4	(28.6%)	1	(7.1%)	3	(21.4%)	0	(0%)	3	(21.4%)	9	(64.3%)
**AAS Only**		**15**	**797** ± 377	**1472** ± 622	**4.35** ± 1.93	**0.53** ± 0.96	**4**	**(26.7%)**	**3**	**(20%)**	**2**	**(13.3%)**	**1**	**(6.7%)**	**4**	**(26.7%)**	**8**	**(53.3%)**
Remifentanil	(13.8%)	8	806	1682	5.05	0.63	3	(37.5%)	2	(25%)	1	(12.5%)	0	(0%)	2	(25%)	5	(62.5%)
Multimodal	(14.3%)	7	786	1233	3.54	0.43	1	(14.3%)	1	(14.3%)	1	(14.3%)	1	(14.3%)	2	(28.6%)	3	(42.9%)
Opioid-Free	(13.3%)	4	575	1052	2.7	0	0	(0%)	0	(0%)	0	(0%)	1	(25%)	0	(0%)	1	(25%)
**TNX Only**		**7**	**1114** ± 907	**1919** ± 1060	**3.17** ± 2.02	**0.86** ± 1.21	**3**	**(42.9%)**	**1**	**(14.3%)**	**2**	**(28.6%)**	**0**	**(0%)**	**2**	**(28.6%)**	**4**	**(57.1%)**
Remifentanil	(1.7%)	1	1000	1967	3.2	1	1	(100%)	0	(0%)	0	(0%)	0	(0%)	0	(0%)	1	(100%)
Multimodal	(12.2%)	6	1133	1911	3.16	0.83	5	(83.3%)	1	(16.7%)	2	(33.3%)	0	(0%)	2	(33.3%)	3	(50%)
Opioid-Free	(20%)	6	1133	1911	3.16	0.83	5	(83.3%)	1	(16.7%)	2	(33.3%)	0	(0%)	2	(33.3%)	3	(50%)
**None**		**51**	**591** ± 345	**1707** ± 674	**4.67** ± 1.99	**0.78** ± 1.92	**19**	**(37.3%)**	**18**	**(35.3%)**	**11**	**(21.6%)**	**4**	**(7.8%)**	**8**	**(15.7%)**	**40**	**(78.4%)**
Remifentanil	(67.2%)	39	575	1688	4.65	0.95	16	(41%)	17	(43.6%)	8	(20.5%)	4	(10.3%)	7	(17.9%)	32	(82.1%)
Multimodal	(24.5%)	12	642	1769	4.72	0.25	3	(25%)	1	(8.3%)	3	(25%)	0	(0%)	1	(8.3%)	8	(66.7%)
Opioid-Free	(16.7%)	5	570	1863	7.92	0	0	(0%)	0	(0%)	2	(40%)	0	(0%)	1	(20%)	2	(40%)
**Total**		**107**	**757** ± 511	**1664** ± 695	**4.39** ± 1.95	**0.81** ± 1.75	**38**	**(35.5%)**	**34**	**(31.8%)**	**24**	**(22.4%)**	**10**	**(9.3%)**	**26**	**(24.3%)**	**76**	**(71%)**
Remifentanil	(100%)	58	649	1659	4.61	0.84	20	(34.5%)	24	(41.4%)	13	(22.4%)	7	(12.1%)	13	(22.4%)	45	(77.6%)
Multimodal	(100%)	49	886	1669	4.13	0.78	16	(32.7%)	10	(20.4%)	11	(22.4%)	3	(6.1%)	13	(26.5%)	31	(63.3%)
Opioid-Free	(100%)	30	894	1675	3.62	0.7	5	(16.7%)	3	(10%)	8	(26.7%)	1	(3.3%)	7	(23.3%)	16	(53.3%)

Only heparin administration was independently related (*p* < 0.05) to adverse outcomes including blood loss, Hb decrease, ICU vasopressor use, haematoma formation, flap loss and the composites surgical and overall complications. No other intervention resulted in statistically significant association to outcomes. N: Number of patients; AAS: AcetylSalicylic Acid; ICU: Intensive Care Unit; TNX: Tranexamic Acid; Blood Loss [calculated]: HbCalc is the calculated blood volume loss using Jaramillo et al. (2020) [18] method and unit conversion; Hb: Haemoglobin concentration; RBC: Red Blood Cells (Packed); ICU: Intensive Care Unit.

## Data Availability

The data presented in this study are available on request from the corresponding author. The data are not publicly available due to ethical restrictions.
